# Sphingomyelin levels in nondipper and dipper hypertensive patients

**DOI:** 10.3892/etm.2013.1455

**Published:** 2013-12-19

**Authors:** HUAN ZHENG, XIAOYUN XIE, NANZI XIE, HUIFENG XU, JUNLING HUANG, MING LUO

**Affiliations:** 1 Geriatrics Department, Tongji Hospital Affiliated to Tongji University, Shanghai 20065, P.R. China; 2 School of Life Science, Center for Evolutionary Medicine and Informatics, Biodesign Institute, Arizona State University, Tempe, AZ 85281, USA; 3 Cardiology Department, Tongji Hospital Affiliated to Tongji University, Shanghai 20065, P.R. China

**Keywords:** sphingomyelin, hypertension, ambulatory blood pressure monitoring, echocardiography

## Abstract

A number of studies have focused on the association between sphingomyelin (SM) levels and atherosclerosis, however, there are few data concerning the correlation of SM with nondipper hypertension. The present study aimed to investigate the correlation between plasma SM levels and nondipper status in patients with hypertension. A total of 200 hypertensive patients were enrolled and divided into two groups according to their ambulatory blood pressure monitoring (AMBP) results: Dipper group (84 patients) and nondipper group (116 patients). All patients were subjected to transthoracic echocardiography examination and laboratory tests. No statistically significant difference was observed between the two groups in terms of basic clinical characteristics. However, the plasma SM levels in the dipper group were significantly lower than those of the nondipper group (41.9±17.5 vs. 96.4±14.3 mg/dl, P=0.003). The left ventricular mass index (LVMI) was higher in the nondipper patients than in the dipper patients and the diastolic function parameters in the nondipper patients were less favorable. Correlation analysis showed that the SM level was negatively correlated with the magnitude of systolic blood pressure (SBP) fall at night (r=−0.42, P<0.01) and diastolic blood pressure (DBP) fall at night (r=−0.31, P<0.01). The nondipper status had contributory effects on hypertensive concentric hypertrophy and diastolic function impairment. In addition, the plasma SM level was associated with a nondipper pattern of hypertension.

## Introduction

Essential hypertension is a complex disease associated with an increased risk of cardiovascular disorders. Twenty-four-hour ambulatory blood pressure monitoring (ABPM) is easily able to detect the circadian blood pressure (BP) pattern of an individual: systolic blood pressure (SBP) and diastolic blood pressure (DBP) show a nocturnal reduction of ≥10% in healthy subjects. Patients whose BP does not decrease during sleep compared with daytime levels are defined as nondippers. Nondipper hypertensive patients have been reported to be at high risk for target organ damage, including stroke, left ventricular hypertrophy, carotid artery disease, microalbuminuria, nephropathy and myocardial infarction (1). Currently, the pathogenesis of nondipper hypertension remains largely unclear in patients without any renal or endocrine pathology. Previous studies have identified differences in several types of serum biochemical concentrations between dipper and nondipper hypertensive patients, including adiponectin (2), serum calcium and phosphate (3) and cystatin C (4). Further clinical investigations are vital for the exploration of the complicated network involved in nondipper hypertension.

Sphingomyelin (SM) is a ubiquitous substance present in cell membranes where cellular processes including signal transduction, membrane trafficking and protein sorting occur (5). Cross-sectional studies have shown an association of high plasma SM levels with subclinical atherosclerosis (6) and clinical coronary artery disease (7). The majority of studies have focused on the association among SM levels, lipid metabolism and atherosclerosis. To the best of our knowledge, the correlation of SM with dipper or nondipper status in hypertension has not yet been studied. The present study was designed to investigate plasma SM levels in patients with dipper and nondipper hypertension.

## Subjects and methods

### Subjects

The study was conducted on patients who attended the outpatient department of Tongji Hospital Affiliated to Tongji University (Shanghai, China). The study participants consisted of 200 consecutive hypertensive patients. BP was measured from the right arm using a standard mercury sphygmomanometer after 10 min of rest with the patient in the sitting position. SBP was measured at Korotkoff phase I and DBP at Korotkoff phase V, following recommendations of the American Heart Association (8). BP was measured three times with an interval of ≥30 sec and the mean values were used for analysis. Hypertension was defined as an SBP of >140 mmHg and/or a DBP of >90 mmHg on repeated measurements and/or receipt of antihypertensive treatment. Exclusion criteria included the presence of the following: Known coronary artery disease (angina and/or electrocardiogram signs of ischemia on treadmill-exercise test), chronic renal failure, chronic liver disorder, moderate or severe valvular disease, diabetes mellitus, congenital heart disease, left ventricular systolic dysfunction on echocardiography (ejection fraction <50%), anemia, thyroid disorder, pregnancy, obstructive sleep apnea, Alzheimer's Disease (it has been revealed that high plasma SM level is associated with Alzheimer's Disease) (9), hyperuricemia and secondary hypertension. All participants provided their informed consent. This study was approved by the Ethics Committee of Tongji Hospital Affiliated to Tongji University.

### Laboratory tests

A venous blood sample was collected from each participant under fasting conditions. Fasting blood glucose, total cholesterol, high-density lipoprotein cholesterol, low-density lipoprotein cholesterol, triglyceride, urea nitrogen, creatinine and uric acid were measured by standard laboratory methods.

Plasma SM levels were measured with an enzymatic method using a four-step procedure according to a previous study (7). In the first step, bacterial sphingomyelinase hydrolyzed SM to phosphorylcholine and N-acylsphingosine. Thereafter, the addition of alkaline phosphatase generated choline from phosphorylcholine. The newly formed choline was used to generate hydrogen peroxide in a reaction catalyzed by choline oxidase. Finally, with peroxidase as a catalyst, hydrogen peroxide was used together with phenol and 4-aminoantipyrine to generate a red quinone pigment with an optimal absorption at 505 nm. The plasma SM levels were measured in a blinded fashion and the interassay coefficient of variation ranged from 2.2 to 4.5%.

### Ambulatory BP recordings

Ambulatory 24-h BP monitoring was performed using a SunTech Oscar2 ABPM recorder (Suntech Medical Inc., Morrisville, NC, USA). Automatic BP recordings were obtained every 30 min during the 24-h period. The cuff was placed around the non-dominant arm of the subjects. Daytime was defined as 7:00 a.m. to 11:00 p.m. and nighttime was defined as 11:00 p.m. to 7:00 a.m. The percentage of nocturnal BP dipping was calculated using the following formula: 100 × [1-(nighttime SBP/daytime SBP)]. A nocturnal BP dip was defined as a >10% reduction in both nocturnal SBP and DBP compared with the average day-time BP. Detection of a <10% reduction in either SBP or DBP was regarded as nondipper hypertension.

### Transthoracic echocardiography examination

All participants underwent complete transthoracic echocardiographic studies (Vivid 7 system; General Motors Co., Detroit, MI, USA), including two-dimensional, color flow and spectral Doppler imaging, using a 2.5–4.0 MHz transducer. An electrocardiograph was recorded simultaneously for every subject. Echocardiographic measurements were obtained with participants in the left lateral decubitus position. Three consecutive cycles were averaged for each parameter. The examinations were performed by an experienced cardiologist who had no knowledge of the participant's clinical information.

Standard views, including the parasternal long-axis, short-axis at the papillary muscle level, apical four-chamber and two-chamber views were recorded. Left atrial volume index (LAVI; left atrial volume divided by body surface area), left ventricular end-diastolic diameter (LVDd), left ventricular end-systolic diameter (LVSd), interventricular septum thickness in diastole (IVST), posterior ventricular septum thickness in diastole (PVST) and left ventricular mass index (LVMI; left ventricular mass divided by body surface area) were collected. The left ventricular mass (LVM) and body surface area (BSA) were calculated using the formula (10): LVM (g) = 1.04[(IVST + LVDd + PVST)^3^ − (LVDd)^3^]− 13.6 and BSA (kg/m^2^) = 0.06 × height + 0.0128 × weight − 0.1529. M-mode tracing of LV was obtained in the parasternal long-axis view with the cursor placed at the tip of the mitral valve leaflets. Left ventricular ejection fraction (LVEF) was estimated using a modified Simpson's biplane method (11).

Pulsed Doppler recordings of mitral flow velocities were obtained from the apical 4-chamber view by placing the sample volume between the tips of the mitral leaflets and LV outflow velocities were obtained by placing the sample volume in the outflow tract below the aortic valve leaflets. Peak early (E) and late diastolic (A) transmitral filling flow velocities, the E/A ratio and the deceleration time (DT) of the E wave were measured. Isovolumic relaxation time (IRT), defined as the time from aortic valve closure to mitral valve opening, was assessed by simultaneously measuring the flow into the LV outflow tract and mitral inflow by Doppler echocardiography.

### Statistical analysis

Statistical analyses were made using SPSS (Statistical Package for the Social Sciences version 15, SPSS Inc., Chicago, IL, USA) software. Numerical variables are presented as mean ± standard deviation and categorical variables are presented as percentage values. The Student's t-test was used for group comparisons. The Mann-Whitney U test was used for comparison of abnormally distributed data. Categorical data were compared with the χ^2^ test. Pearson correlation was used to evaluate the association between SM levels and demographics or laboratory parameters. P<0.05 was considered to indicate a statistically significant result.

## Results

According to 24-h ABPM monitoring, dipper and nondipper hypertension was noted in 84 patients (42%) and 116 patients (56%), respectively. Comparisons of clinical and biochemical variables in the dipper and nondipper groups are shown in Table I. There was no statistically significant difference between the two groups in terms of age, gender distribution, body mass index (BMI), smoking status or antihypertensive medications. The concentrations of total cholesterol, high density lipoprotein, low density lipoprotein, triglyceride, fasting glucose, urea nitrogen and creatinine were similar in the two groups. The SM levels were significantly lower in the dipper group than in the nondipper group (41.9±17.5 vs. 96.4±14.3 mg/dl, P=0.003).

The ABPM parameters of the patients are summarized in Table II. No significant differences were identified in 24-h mean SBP, between the two groups. However, mean nighttime SBP and mean nighttime DBP were lower in the dipper group than in the nondipper group (118.2±13.3 vs. 129.4±12.7 mmHg and 71.6±7.0 vs. 79.1±7.2 mmHg, respectively; both P<0.001). Additionally, the rates of SBP and DBP fall in the nighttime were clearly different between the two groups (12.8±6.4 vs. 1.1±5.8% and 11.4±5.2 vs. 0.8±6.1%, respectively; both P<0.001).

The results of the echocardiographic examinations are shown in Table III. No significant differences in LVSd, LVDd, IVST, PVST or LVEF were identified between the two groups, but LAVI, LVMI, DT and IRT were higher in the nondipper group than in the dipper group (26.5±4.6 vs. 23.2±3.6 ml/m^2^, P=0.02; 122.8±12.1 vs. 108.9±14.6 g/m^2^, P=0.007; 234.9±19.5 vs. 211.3±25.4 msec, P=0.03; and 100.1±7.3 vs. 85.7±8.2 msec, P=0.02, respectively), while the E/A ratio was lower in the nondipper group than in the dipper group (0.74±0.21 vs. 0.91±0.13, P=0.009).

**Figure 1:**
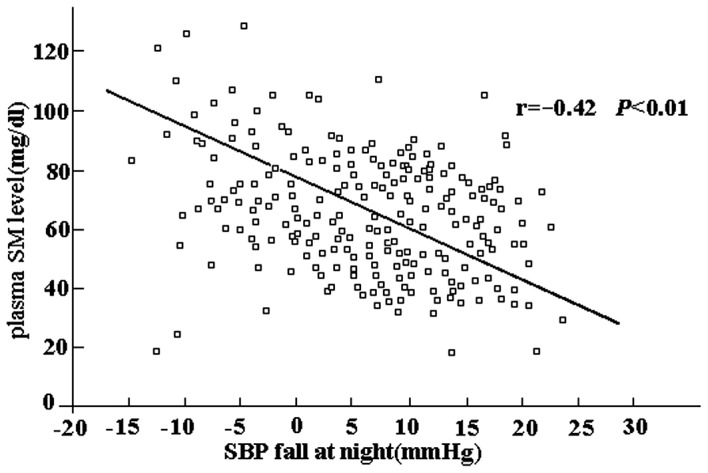
Linear regression curve of the correlation between plasma SM levels and SBP fall at night in hypertensive patients. SM, sphingomyelin; SBP, systolic blood pressure.

**Figure 2:**
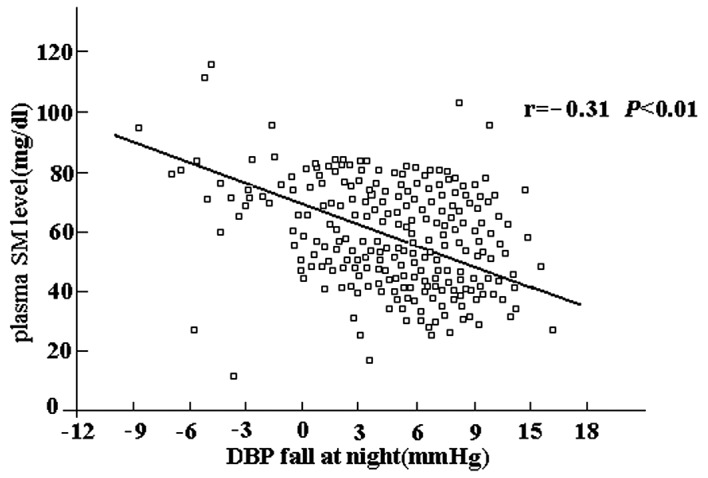
Linear regression curve of the correlation between plasma SM levels and DBP fall at night in hypertensive patients. SM, sphingomyelin; DBP, diastolic blood pressure.

In correlation analyses, the plasma SM level was identified to be negatively correlated with the magnitude of SBP fall at night (r=−0.42, P<0.01; Fig. 1) and DBP fall at night (r=−0.31, P<0.01; Fig. 2). In addition, the SM level was correlated with age (r=0.39, P=0.02), BMI (r=0.25, P=0.01) and low density lipoprotein (r=0.43, P<0.01).

## Discussion

In this study, it was demonstrated that the LVMI and left ventricular diastolic parameters were less favorable in the nondipper hypertensive group compared with the dipper group. In addition, plasma SM levels were significantly increased in nondipper hypertensive patients compared with those in dipper hypertensive patients. Furthermore, plasma SM levels were negatively correlated with the fall in SBP and DBP at night. To the best of our knowledge, this is the first study to investigate the association between plasma SM levels and the BP nondipper pattern.

A 24-h ABPM is an effective, reliable, noninvasive and inexpensive method for BP ambulatory measurement and circadian rhythm determination. Based on the ABPM results, hypertension may be easily classified as dipper or nondipper pattern. The nondipper pattern has been demonstrated to be associated with an increased risk of cardiovascular, cerebrovascular and renal complications (1,12,13). It is generally acknowledged that transthoracic echocardiographic examination is also an effective and reliable method for hypertensive heart follow-up. A tissue Doppler study reported that the nondipper pattern had an influence on increased LVM, impaired left ventricular systolic and diastolic dysfunction and higher left ventricular filling pressures in hypertensive patients (14). In addition, in a recent study, left atrial appendage filling and ejection flow rates were observed to be decreased in nondipper hypertensive patients compared with those in dipper hypertensive patients and control subjects, which is likely to cause left atrial appendage dysfunction (15). In the present study, the echocardiographic findings for dipper and nondipper hypertensive groups were consistent with certain previous results. LVMI in the nondipper hypertensive group was higher than that in the dipper group, suggesting that nondipper status had contributory effects to hypertensive left ventricular concentric hypertrophy. In addition, the left ventricular diastolic parameters in the nondipper group were less favorable than those in the dipper group. Left ventricular diastolic dysfunction may develop much earlier than left ventricular hypertrophy and systolic dysfunction in hypertensive patients. The duration of diastole is an important determinant of myocardial perfusion. Researchers who have obtained similar results concerning nondipper hypertensive patients having a less favorable cardiac performance have proposed the adoption of more aggressive antihypertension treatment for those patients (14–16).

Although the detrimental impacts of the nondipper BP pattern have been studied extensively among hypertensive patients, its exact mechanisms of action have not yet been elucidated. It has been suggested that nondippers display impaired autonomic dysfunction (17), higher sympathetic activity (18), higher inflammatory activity (19,20), prominent insulin resistance (2) and increased oxidative stress leading to endothelium-dependent vasodilation dysfunction (21). Clinical observations have shown the association of several plasma biomarkers with nondipper hypertension, including plasma atrial and brain natriuretic peptides (22) and vitamin D deficiency (23). However, there are few data concerning lipoprotein metabolism and nondipper hypertension. SMs, once considered mainly to be structural components of cell membranes, have emerged as key signaling molecules involved in a range of cellular functions, including cell growth and differentiation, proliferation and apoptotic cell death (24). An increasing amount of evidence shows that plasma SM level is an independent risk factor for atherosclerosis that shares common features with hypertension, including enhanced oxidative stress, chronic inflammatory responses and altered endothelial and vascular muscle smooth cell functions. In an epidemiological case-control study, Jiang *et al* observed that the plasma SM level was positively and independently correlated with age, BMI and SBP (8). Nelson *et al* then investigated whether plasma SM was an early atherogenic risk factor and examined the associations between plasma SM level and carotid intimal-medial wall thickness, ankle-arm BP index and the Agatston coronary artery calcium score in asymptomatic adults. Nelson *et al* concluded that plasma SM was associated with subclinical atherosclerotic disease (6). In the present study, it was observed that plasma SM levels were higher in the nondipper hypertensive group than in the dipper group, and were negatively correlated with the fall in SBP and DBP at night. The present study is a clinical observational study and did not investigate the mechanism by which SM levels were increased in nondipper hypertensive patients. The identification of mechanistic pathways is extremely important for improving the understanding of nondipper hypertension and its treatment. As a previous study has identified that SM biosynthesis is tightly linked to development of insulin resistance, obesity and atherosclerosis (25), we suggest that SM acts as a link between several key chains, including insulin resistance, mediation of cell proliferation and oxidative stress, involved in a complicated mechanistic network in nondipper hypertension.

In conclusion, the present study indicated that the nondipper pattern had contributory effects on hypertensive concentric hypertrophy and diastolic functional impairment. In addition, the plasma SM level was associated with the nondipper pattern in hypertensive patients. The measurement of SM may be used to indicate an increased risk of nondipper hypertension-associated adverse cardiovascular events. The major limitation of this study is its relatively small sample size and lack of a control group. Further large-scale studies are required to assess the effects of SM on the development of nondipper hypertension.

## Figures and Tables

**Table I:** Comparisons of the clinical and biochemical variables between dipper and nondipper hypertensive patients.

Variable	Dipper hypertensive patients (n=84)	Nondipper hypertensive patients (n=116)	P-value
Age (years)	57.3±8.4	58.6±9.1	0.740
Gender, M/F (n)	40/44	49/67	0.260
BMI (kg/m^2^)	27.8±3.3	26.9±4.2	0.360
Smoking history (n, %)	30 (35.7)	37 (31.9)	0.400
ACEI-ARB (n, %)	47 (60.0)	66 (56.9)	0.560
CCB (n, %)	34 (40.5)	54 (46.6)	0.530
β-blocker (n, %)	9 (10.7)	10 (8.6)	0.280
Diuretics (n, %)	7 (8.3)	9 (7.8)	0.610
Total cholesterol (mg/dl)	208.1±22.6	199.7±25.4	0.370
HDL (mg/dl)	40.6±12.1	41.2±11.8	0.460
LDL (mg/dl)	122.6±27.8	115.9±30.4	0.130
Triglyceride (mg/dl)	186.5±31.0	192.6±28.2	0.620
Fasting glucose (mg/dl)	98.7±10.4	101.3±12.5	0.270
Urea nitrogen (mg/dl)	26.4±8.2	28.7±7.1	0.800
Creatinine (mg/dl)	0.82±0.13	0.86±0.17	0.780
SM (mg/dl)	41.9±17.5	96.4±14.3	0.003

Values are mean ± SD or numbers (percentage). BMI, body mass index; ACEI, angiotensin converting enzyme inhibitor; ARB, angiotensin II receptor blocker; CCB, calcium channel blocker; HDL, high density lipoprotein; LDL low density lipoprotein; SM, sphingomyelin.

**Table II:** Comparison of ambulatory blood pressure monitoring results between dipper and nondipper hypertensive patients.

Variable	Dipper hypertensive patients (n=84)	Nondipper hypertensive patients (n=116)	P-value
24-h mean SBP (mmHg)	132.6±12.8	133.7±11.9	0.460
24-h mean DBP (mmHg)	78.3±11.6	81.5±10.1	0.370
Mean daytime SBP (mmHg)	137.9±10.4	136.8±10.2	0.510
Mean daytime DBP (mmHg)	84.8±8.3	83.0±9.5	0.250
Mean nighttime SBP (mmHg)	118.2±13.3	129.4±12.7	<0.001
Mean nighttime DBP (mmHg)	71.6±7.0	79.1±7.2	<0.001
Rate of SBP fall at nighttime (%)	12.8±6.4	1.1±5.8	<0.001
Rate of DBP fall at nighttime (%)	11.4±5.2	0.8±6.1	<0.001

Values are mean ± SD; SBP, systolic blood pressure; DBP, diastolic blood pressure.

**Table III:** Comparison of echocardiographic parameters between dipper and nondipper hypertensive patients.

Variable	Dipper hypertensive patients (n=84)	Nondipper hypertensive patients (n=116)	P-value
LAVI (ml/m^2^)	23.2±3.6	26.5±4.6	0.020
LVDd (mm)	46.3±4.9	45.9±5.5	0.260
LVSd (mm)	28.9±3.1	29.1±4.0	0.370
IVST (mm)	11.2±1.6	11.3±1.5	0.590
PVST (mm)	10.9±1.3	10.6±1.4	0.470
LVMI (g/m^2^)	108.9±14.6	122.8±12.1	0.007
LVEF (%)	64.3±6.8	62.4±7.1	0.160
E/A	0.91±0.13	0.74±0.21	0.009
DT (msec)	211.3±25.4	234.9±19.5	0.030
IRT (msec)	85.7±8.2	100.1±7.3	0.020

Values are mean ± SD. LAVI, left atrial volume index; LVDd, left ventricular end-diastolic diameter; LVSd, left ventricular end-systolic diameter; IVST, interventricular septum thickness in diastole; PVST, posterior ventricular septum thickness in diastole; LVMI, left ventricular mass index; LVEF, left ventricular ejection fraction; E/A, peak early and late diastolic transmitral filling flow velocities ratio; DT, deceleration time of the E wave; IRT, isovolumic relaxation time.
